# Responses to dying and dead adult companions in a free-ranging, provisioned group of Japanese macaques (*Macaca fuscata*)

**DOI:** 10.1007/s10329-025-01196-2

**Published:** 2025-06-24

**Authors:** M. Nakamichi, K. Yamada

**Affiliations:** Graduate School of Human Sciences, Osaka University, 1-2 Yamadaoka, Suita, Osaka 565-0871 Japan

**Keywords:** Social affinity, Grooming, Proximity, Aversion to maggots, Awareness of death

## Abstract

We report behavioral responses to four dying and dead adult companions (two aged alpha males, one high-ranking adult male, and one aged, high-ranking female) in a free-ranging, provisioned group of Japanese macaques, with particular interest in whether external, visible bodily damage to the deceased individuals and social affinity with them influenced responses of group members. All but one moved away or stayed away from the dying or dead individuals when they noticed maggots on them, likely indicating an aversion to maggots (Cases 1, 2, and 3). However, some individuals remained near the corpse of an adult male that had no noticeable external damage, and a juvenile female whose mother was his most frequent proximity partner groomed him (Case 4). One adult female who ate maggots while grooming the dying alpha male (Case 1) and most individuals who remained relatively close to another alpha male’s rain-drenched dead body (Case 2) were also affiliated with those two alpha males prior to their deaths. We conclude that Japanese monkeys have a strong tendency to avoid dying and dead adult companions already infested with maggots but that some individuals with social affinity with the deceased may contact or at least remain near the latter for some time. We discuss monkeys’ possible perceptions when encountering corpses, in relation to non-human primates’ awareness of death.

**Supplementary Information:**

The online version contains supplementary material available at 10.1007/s10329-025-01196-2.

## Introduction

Many mothers of various non-human primate species ranging from lemurs to apes respond toward their dead infant in diverse ways such as sniffing, licking, touching, holding, grooming, and returning to the corpse. In many but not all of those species, the dead infant may also be carried for some time (see reviews by Fernández-Fueyo et al. 2021; Watson and Matsuzawa 2018). In contrast, responses to dead bodies of adult group members are scarcely reported (Gonçalves and Carvalho 2019), and those are taxonomically biased toward great apes (Anderson 2018). Minami and Ishikawa (2023) were able to retrieve only 45 studies describing adult deaths in non-human primate species (31 for great apes, 14 for monkeys), most of which included non-contact actions such as staying near and looking at the corpse, and in two thirds of which at least one group member physically contacted the corpse, most often touching it briefly (see Supplementary Table 1 of Minami and Ishikawa 2023).

Human emotions and behaviors in response to death vary greatly depending on an individual's relationship with the deceased prior to their death. This may be true also for non-human primates who live in complex social groups with individual recognition and long-term affiliative relationships with specific group members (De Marco et al. 2020, 2022; Gonçalves and Carvalho 2019; van Leeuwen et al. 2016). Similar to when a dead infant is carried by its mother who has unquestionably the closest social bond with it, the corpse of an older individual may elicit responses particularly by those who were close to that individual when alive. How social bonds influence non-human primates’ responses to the deceased is one of the fundamental questions for our understanding of phylogenetic continuity between human and non-human primate thanatological responses.

External conditions of the corpse may influence how conspecifics respond. Victims of disease may have few overt external wounds, whereas those killed by predators, lethal intergroup attacks, or traffic accidents may have severely and visibly damaged bodies (Campbell et al. 2016). Moreover, open wounds on the skin may fester and attract flies. The latter lay eggs and, depending on climatic conditions, the maggots exacerbate deterioration of a dying individual’s external body condition. Maggots may influence how group members respond to dying and dead individuals.

Japanese macaques (*Macaca fuscata*) have been studied in detail over a long period in the wild, free-ranging but provisioned, and captive conditions; thus, their social structures and social behaviors are relatively well documented (e.g., Nakagawa et al. 2010). However, only four reports describe responses of group members to dead adult conspecifics (Iida 1999; Kawai 1960; Mizuhara 1976; Minami and Ishikawa 2023). The most recent report provided detailed information, including who contacted the corpse of an adult female and what behaviors occurred, taking into account prior social affinity with the dead female. Only one non-affiliated adult female groomed the freshly dead female’s body, which showed no external damage (Minami and Ishikawa 2023). More detailed descriptions about who responds to adult corpses and how are needed for a fuller understanding of thanatological behavior in non-human primates.

In this paper, we report four cases of responses to dying and dead adult group members in a free-ranging, provisioned group of Japanese macaques, at Katsuyama, Japan. Over several years we recorded grooming interactions and proximity relationships among adult individuals of this group, to elucidate their long-term affiliative relationships. Using these grooming and proximity data, commonly considered as powerful behavioral measures of social affinity between individuals (Gouzoules and Gouzoules 1987; Stoinski et al. 2003), we examined whether individuals behave differently toward four dying and dead conspecifics depending on pre-death social affinity. The corpse of one individual who died in winter had almost no visible injuries and was maggot-free, whereas the bodies of three others who died in summer were already attracting flies and were maggot-infested before death. Thus, we also examined how the external condition of dying and dead individuals influenced responses of group members toward them.

## Methods

M.N. observed all four cases and K.Y. one while observing the Katsuyama free-ranging, provisioned group of Japanese macaques, Okayama Prefecture, Japan, in and around the group’s feeding site, for their respective research projects. The group has been artificially provisioned and individual identification on all group members has been maintained since 1958. The group size varied from 154 to 207 individuals, including 6–15 adult males (6 years of age or more) and 64–93 adult females (5 years of age or more) in April of four observation years (1993, 1999, 2003 and 2007). More information on the group and the feeding site is described elsewhere (Nakamichi and Yamada 2024). We used ad libitum notetaking to record the four thanatological cases described in the Observations section. Some qualitative descriptions of Cases 2 and 3 have been reported in Japanese (Nakamichi 2019).

From 1990 to 2015, M.N. routinely recorded names of adult individuals who groomed each other in 20-min sessions (hereafter, 20-min grooming session) and those who were within 2 m and 5 m from central adult males in 20-min sessions (hereafter, 20-min proximity session), albeit with some interruptions (see Nakamichi and Shizawa 2003 and Nakamichi et al. 1995 for detailed observation procedures for grooming and proximity, respectively). As grooming in Japanese macaques is concentrated within a small subset of available partners (Nakamichi and Shizawa 2003), we considered two individuals with recorded grooming interactions to have a social affinity. Arbitrarily, we also considered the 10 individuals most frequently in proximity (within 5 m) to a given central male as having a social affinity with him. Based on these criteria, we examined whether individuals who responded to dying or dead group members had a social affinity with them before death.

We performed a Chi-square test and Fisher’s exact test to evaluate behavioral changes between two periods before death in Case 1. Statistical significance was set at *p* < 0.05.

## Observations

### Case 1. Responses to a dying, aged alpha male with a maggot-infested wound

*Rikinio* (formal name: *M65 Rika’60’*) maintained his alpha position for 17 years until just before his death at 28 years of age in August, 1993. For about one month before his death, from July 1, 1993, he stayed in the vicinity of the feeding site not only during the day but also at night, due to his diminished locomotor ability. When the group was there, he interacted with group members; his alpha position remained clear. On August 3, he was suddenly attacked by the second-ranking male and bitten on his back. At the time, he received agonistic support from the 22-year-old alpha female, *Pet* (formal name: *F71 Bera’53’*). The wound resulting from the bite soon festered, and *Rikinio’s* physical condition deteriorated. On the fourth day after the bite (August 7, his last day in the group), several flies were resting or swarming around the wound, which was several cm long, and in and around which maggots were visible, but he was still able to walk, albeit with much difficulty. The following morning, *Rikinio* was found alone on a sidewalk for tourists around 350 m away from the feeding site, unable to move; he was put inside a cage and the cage was placed indoors by park staff. He died 3 days later (see Nakamichi et al. 1995 and Nakamichi 2019 for detailed information on his dominance relations with the second-ranking male and related events).

In the month before *Rikinio* was attacked, 66% of his 47 interactions involving physical contact with other group members were initiated by the latter, but on his last day, they initiated only 30% (*X*^*2*^(1) = 7.680, *p* < 0.01: Table 1). Furthermore, 71% of 35 observed interactions were terminated by the other individuals in the month prior the attack, compared to 96% on his last day (Fisher’s exact test, *p* = 0.065: Table 1). These findings show that some group members continued to initiate contact with the declining *Rikinio* in the month before he was attacked; however, they clearly avoided physical contact with him when he was infested with maggots on his last day in the group.

**Table 1:** The distribution of iniciators and terminators of social interactions involving physical contact such as grooming between *Rikinio* and other group members in the month before he was attacked and on his last day in the group

		In the month before *Rikinio* was attacked^1)^	On *Rikinio*’s last day in the group (3 days before his death)^2)^	Test
Initiator	*Rikinio*	16 (34.0%)	19 (70.4%)	*X*^*2*^(1) = 7.680, *p* < 0.01
	Other individuals	31 (66.0%)	8 (29.6%)	
Terminator	*Rikinio*	8 (22.9%)	1 (3.8%)	Fisher’s exact test, *p* = 0.065
	Other individuals	27 (77.1%)	25 (96.2%)	

^1)^During four observation days in the month from July 1 to August 3, 1993, before *Rikinio* was attacked

^2)^August 8, 1993

**Fig. 1:**
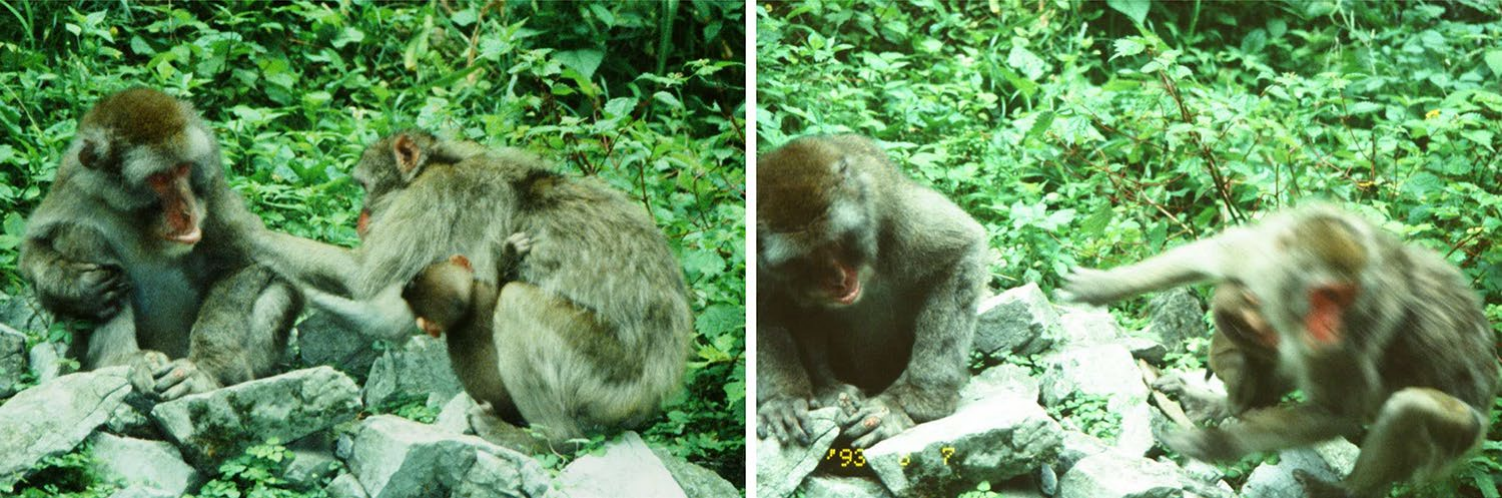
(Left) A 9-year-old, high-ranking female (*Mara68′94*) starts grooming *Rikinio* on his last day with the group (10:49, August 7, 1993). (Right) Around 10 s later, upon bringing her hand close to the wound on his back (probably touching the maggots), she immediately jumps back (see Table 1, no. 4)

**Fig. 2:**
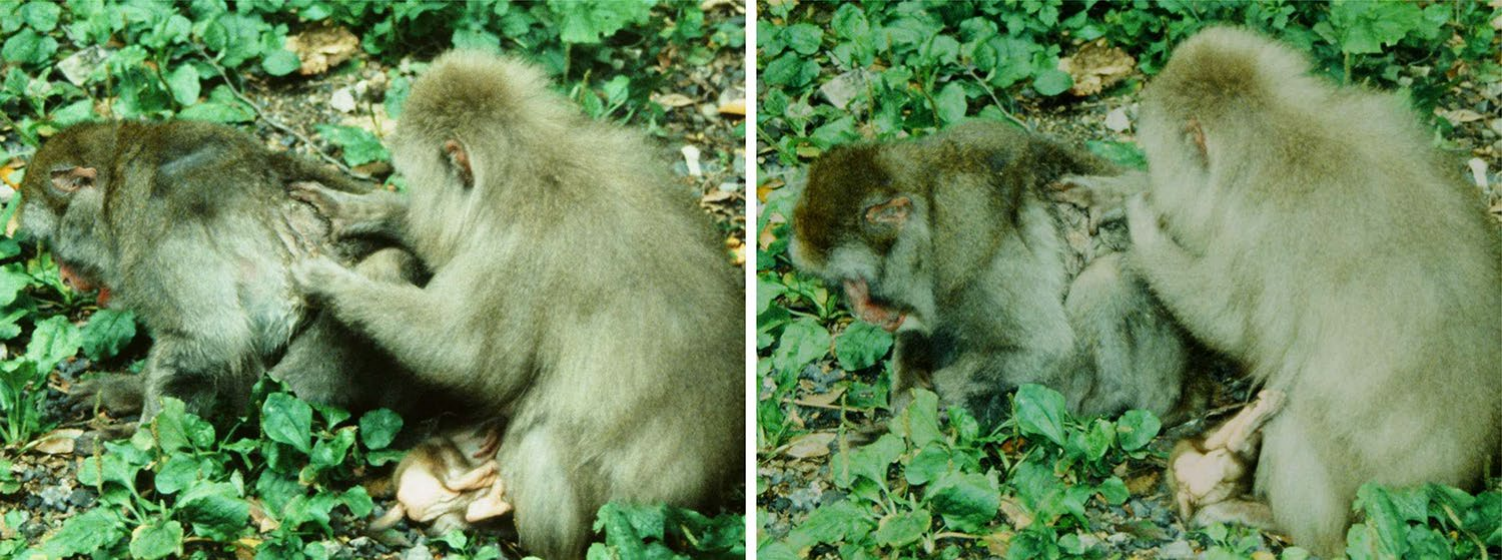
(Left) *Pet* (22-year-old alpha female) picks up a maggot between her thumb and forefinger when grooming around the wound on the left side of *Rikinio*’s back. (Right) She brings the maggot to her mouth to eat (14:42, August 7, 1993) (see Table 1, no. 12)

On his last day in the group, nine individuals (1 juvenile male, 3 juvenile females and 5 adult females) were observed to move away from *Rikinio* a total of 10 times after looking at, sniffing, or touching the maggots on his back (Table 2). In seven of the 10 cases they jumped back or ran away immediately after sniffing or touching the maggots, in a clearly aversive response (Fig. 1 and Table 2). Of the five adult females who showed aversive responses, four had groomed *Rikinio* during the 4-month period prior to him being attacked (Nos. 1, 4, 5, and 6 of Table 2). By contrast, the alpha female (*Pet*), *Rikinio*’s primary adult grooming and proximity partner in the four months before the attack, groomed his wound three times, picking up and eating maggots twice; she showed no aversive response on his last day (Fig. 2 and Nos. 10, 12, & 13 of Table 2). Another five individuals (2 juvenile females, 1 unidentified juvenile male, and 2 adult females) were also observed to groom *Rikinio* on his last day without displaying any aversive response. Instead of grooming the maggot-infested wound, these individuals focused on other body parts (probably they did not notice the maggots). In fact, one female (No. 5 of Table 2) groomed the right side of his back for 9 min, but as soon as she touched the maggots on the left side of his back, she jumped backwards and moved away.

**Table 2:** Reactions of group members to maggots adhering to the wound on *Rikinio*’s left side of his back on the last day he specnt with the group (Aug. 7, 1993)

No	Time	Name*	Sex	Age	Dominance rank	Interactions with *Rikinio* for four months befor death		Reaction to maggots	Notes
						Grooming^1)^	Proximity^2)^		
1	8:35	*Pet83*	f	10	High	Yes	4th	Aversive	She approached to within 2 m and looked at his back, then left
2	8:51	*Pet92*	f	1	High			Aversive	About 30 s after starting grooming, she looked at maggots and left
3	9:05	*Elzia73′92*	m	1	High			Aversive	Within 1 min after making contact with him, this juvenile male sniffed his back and left
4	10:49	*Mara68′84* (1)	f	9	High	Yes	5th	Aversive	Around 10 s after she started grooming him, as soon as her hand moved near to the wound, she immediately jumped back. (see Fig. 1)
5	10:50	*Lipkira72′85*	f	8	Low	Yes	9th	Aversive	She groomed the right side of his back for around 9 min, occasionally brushing flies away with her hand, but immediately jumped back when her hand touched the maggots near the wound on his left side of his back
6	11:06	*Masia72*	f	21	High	Yes		Aversive	She sniffed the magotts and then jumped back the moment she touched the maggots
7	11:10	*Tera68′77′86*	f	7	Middle			Aversive	She sat down nearby and jumped back the moment she touched his back
8	12:15	*Mara68′84* (2)	f	9	High	Yes	5th	Aversive	After bringning her face to around 10 cm from his back and looking at the maggots, she ran away
9	12:20	*Pet'83′89*	f	4	High			Aversive	She approached and brought the tip of her nose to the maggots, about 10 cm, and immediately ran away
10	12:25	*Pet* (1)	f	22	α female	Yes	1st	Positive	While grooming near his wound she touched the maggots on his back for around 30 s, and then she left as her infant was picked up by another individual
11	12:25	*Pet79′89*	f	4	High			Aversive	She looked at and sniffed the maggots, then immediantely ran away
12	14:40	*Pet* (2)	f	22	α female	Yes	1st	Positive	While grooming him, she took maggots on his back and ate them. (see Fig. 2)
13	15:35	*Pet* (3)	f	22	α female	Yes	1st	Positive	While grooming him, she took maggots on his back and ate them for at least 2 min

*(1), (2) and (3) written after names indicate that it is the first, second, and third observations of the individual’s involvement with the maggots on Rikinio’s back, respectively

^1)^Adult individuals who were observed to have grooming interactions with *Rikinio* at least once in 267 20-min grooming sessions conducted during the 4-month-period before his death

^2)^Adult individuals included in the top 10 animals with the highest frequency of proximity to *Rikinio* during the 4-month-period before his death. Ordinal numbers reveal the order of the individuals. The number of 20-min proximity sessions conducted during this period was 184. In 179 of these 184 sessions *Rikinio* was observed. For example, *Pet, Rikinio*’s primary proximity partner, was observed in 27 (15.1%) of the 171 20-min proximity sessions, and *Pet83,* his fourth most frequent proximity partner, was in 16 (8.9%)

### Case 2: Responses to a dying and dead aged alpha male

*K75* (formal name, *M75 Kera’55′61’*) maintained his alpha position for 6 years until his death on August 9, 2003 at 28 years of age. Although his physical condition deteriorated gradually due to old age, he traveled in the forest together with the group until three days before his death. *K75* had grooming interactions with adults (23 females and 1 male) in the 4 months before his death (Fig. S1 of Supplementary Information), indicating continuing affiliative relationships with some group members.

Three days before death (August 6, 2003), *K75* was noticeably struggling to walk. He was groomed once, by the 32-year-old alpha female (*Pet*), who was his most frequent grooming partner in the four months before his death, but apparently not by anyone else. He remained alone at the feeding site after the group left. From then on until his death, he did not leave the feeding site. The following day (August 7), the group did not enter the feeding site. *K75’s* right forearm and leg were swollen and some flies began swarming around and landing on his body, although there was no noticeable wound. The day before *K75*’s death (August 8), the group entered the feeding site in the morning, and left around noon. While at the site, no one approached *K75*, who hardly moved*,* but sat alone all day. In the evening, at least 10 flies with larvae were seen on his forehead.

At 08:00 on August 9, we found *K75* lying dead a few meters away from where we last saw him the evening before. As his forehead was covered with grass, we did not know if any maggots were on it. The body was drenched from rain the previous night. The group was already in the vicinity of the feeding site, but all members except four adult females were still on the other side of the river, 20 m or more away from the dead body. Usually, the monkeys descended the steep slope then quickly crossed the river to enter the feeding site; the reluctance of most of them to cross the river and instead remaining on the opposite bank suggested avoidance of the corpse.

The four females who had already crossed the river and were now 10–15 m away from *K75* included the alpha female *Pet*, *K75*’s youngest sister (*Keriia’84*, 19 years old) and her adult daughter (*Keriia’84′96*, 7 years old) (Fig. S2). These three had grooming interactions or were frequently in proximity to *K75* in the four months before his death, but the fourth female (*Vivia’72′83*, 10-year-old) was not seen either grooming or in proximity with him (Table S1). None of the four showed any overt response to *K75*’s dead body; no looking at it or approaching.

When soybeans and wheat were scattered near the corpse of *K75*, *Pet* started to approach him, but she stopped 2–3 m away and looked at him for a few seconds, before running back several meters, stopping, looking back, and running farther away. Then, a 7-year-old adult female (*Pet88′96*), another groomer of *K75* before his death and with a clinging infant on her ventrum, came to within around 3 m of the corpse to pick up food, but not closer than that. Several juveniles also approached, but never any closer than 5–6 m. By contrast, a 2-year-old, unrelated female (*Tera68′73′92′01*) approached the corpse to within 1 m and picked up food without showing any sign of aversion (Fig. S3 and Table S1). She had a long-term affiliative relationship with *K75*, who had displayed “male care” toward her (holding, carrying, grooming, etc.) for the past 1.5 years (see Nakamichi et al. 2021). At around 08:40 when there were no individuals within around 20 m of the corpse, it was put into a bag and removed by park staff; no group members reacted to this, being seemingly unaware of it. Soon thereafter, feeding began, and most group members crossed the river to pick up wheat. Some monkeys picked up wheat up to 1 m from where the corpse had lain, but none approached to pick up wheat at that specific spot (Fig. S4).

### Case 3: Aversive responses to a dying, aged female with maggots

A 28-year-old female (*Pet79,* formal name, *F79 Bera’53′71’*), her four daughters (20, 11, 9 and 7 years old) and two younger sisters (19 and 15 years old) were all members of the highest-ranking kin-group in the summer of 2007. *Pet79* was subordinate to her two younger sisters, but dominant to her daughters and all unrelated females.

On July 6, 2007, about one month before her death, *Pet79* returned to the group after 8 or 9 days of absence. Her temporary disappearance from the group was a first for her, and the reason was unknown. For two hours after she rejoined the group, she groomed all four of her daughters in turn and was groomed by her youngest sister. During the year preceding her temporary disappearance, *Pet79* had grooming interactions with 16 adult females, especially her youngest daughter (7 years old) and, to lesser extents, her third and second daughters, but she was never observed grooming with either her first daughter or youngest sister (Table S2). During the four weeks from her return between July 7 and August 2, she groomed again mostly with her youngest daughter (Table S2).

One month after her return (August 7), *Pet79* suffered wounds on her back etc., probably resulting from an attack by an unknown adult male while alone at the feeding site (why she was alone is unknown), and disappeared again. Four days later (August 11), she walked alone into the feeding site (at 12:50). A wound several cm long was visible on the right side of her back, so severe that muscles were exposed. Flies were flying around, landing on her, and many small maggots (around 3 to 5 mm) were seen in and around the wound. Around 20 min later, the group entered the feeding site, and feeding started soon thereafter (at 13:20). As always, *Pet79* and her youngest daughter picked up wheat and ate it in close proximity to each other. After feeding, the youngest daughter started to groom *Pet79*, but when she inspected the wound on her mother’s back, she sniffed her fingers and ran away (Table 3, no.1). *Pet79*’s third daughter also ran to about 10 m away from her about 10 s after starting to groom her back. When she stopped running, she smelled her fingers then rubbed them on the ground (Table 3, no. 2). These behaviors of the two daughters clearly indicated a dislike of maggots. Except for these two daughters, on that day no other group member was seen to groom or sit in contact with *Pet79*, whereas three adult females (her two younger sisters and one unrelated adult female) and one adult male (her nephew and the alpha male) clearly avoided her (Table 3, nos. 3–6). In the evening, she left the feeding site alone.

**Table 3:** Aversive responses of group members to the 28-year-old female *Pet79* with flies and maggots around the wound on her back

No	Time	Information on individuals who responded to *Pet79*					
		Name	Sex	Age	Domi-nance rank	Kinship with *Pet79*	Aversive responses to *Pet79*
August 11, 2007 (the day before *Pet79* could not move at all)							
1	13:50	Pet79′00*	f	7	High	Youngest (fourth) daughter	After starting to groom, she looked at the wound, sniffed her fingers, then ran away. (Hayashi, personal communication)
2	15:48	Pet79′98*	f	9	High	Third daughter	About grooming for 10 s, she sniffed her fingers, ran away about 10 m, sniffed her fingers again, and rubbed them on the ground
3	15:52	Pet92*	f	15	α female	Youngest sister	Immediately upon looking at *Pet79* about 10 m, she ran more than 30 m away
4	15:56	*Brisa71′84′98*	f	9	Middle	Unrelated	After approaching to within 3 m she stopped, looked at the wound for a few seconds and then ran away
5	16:00	*Pet88′00*	m	7	α male	Nephew	He watched from 10 m away as an onion was placed in front on *Pet79*, but did not come close
6	16:21	Pet88*	f	19	High	Younger sister	Immediately upon seeing *Pet79* moving toward her from 10 m, she retrieved her infant and ran away, carrying it on her back
August 12, 2007 (the day *Pet79* could not move at all)							
7	8:42	Pet79′98* and others	f	9	High	Third daughter	When *Pet79* approached, ran away along with her 1-year-old son, 2-month-old son, and several other individuals
8	8:49	Pet88′98*	f	9	High	Niece	After climbing on a rock about 1 m high and 10 m away, she looked at *Pet79,* then she retrieved her infant and ran away
9	8:53	Vivia72′83′95*	f	12	Low	Unrelated	She stopped along with an unidentified juvenile to look at *Pet79* for a few seconds and then walked away (see Fig. 3)
10	8:57	Pet79′00*	f	7	High	Youngest (fourth) daughter	She climbed up a rock to about 1 m high and 8 m away, looked at *Pet79* for a few second, then moved away
11	9:10	*10 or more individuals*					Upon *Pet79* arriving on the sidewalk all monkeys within 10 m of her ran away in the opposite direction

^*^ Adult females who had grooming interactions with *Pet79* during the one-year period between Aug. 2006 and July 2007

**Fig. 3:**
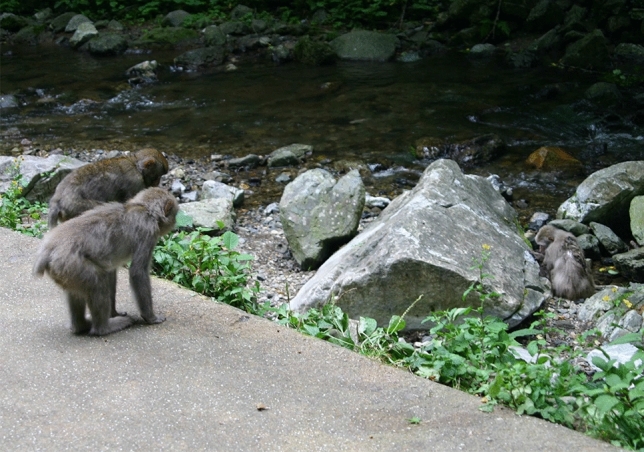
An 12-year-old unrelated female and an unidentified juvenile female stand and look toward *Pet79’*s back. Shortly after, they walked away. Compared to the previous day, there were now many dozens of flies and maggots (around 1 cm in size) around the wound on *Pet79*’s back (08:53 on August 12, 2007)

The following day (August 12), around 30 min after the group entered the feeding site (at 08:20), *Pet79* appeared alone, moving very awkwardly. Her wound appeared to have festered considerably, and dozens of flies and large maggots were on her back, partly covering the wound. When she approached a subgroup of around 10 individuals including her third daughter who were resting or picking up leftover wheat, they all immediately ran away (Table 3, no. 7). Thereafter, no one approached her. Her youngest daughter climbed onto a rock about 1 m high and about 10 m away from *Pet79* and looked at her, then climbed down and walked away (Table 3, no. 10). Other individuals also avoided *Pet79* after staring at the maggots on her back (Fig. 3 and Table 3, nos. 8, 9, & 11). As *Pet79* became unable to move at all on the sidewalk at the feeding site and looked close to death, she was moved to another area of the park by staff at 10:00: she was put into a small box which was then put on the trailer of a nearby car; this was done out of view of any group members. No information is available on what happened to *Pet79* next.

### Case 4: Responses to an intact adult male corpse

**Fig. 4:**
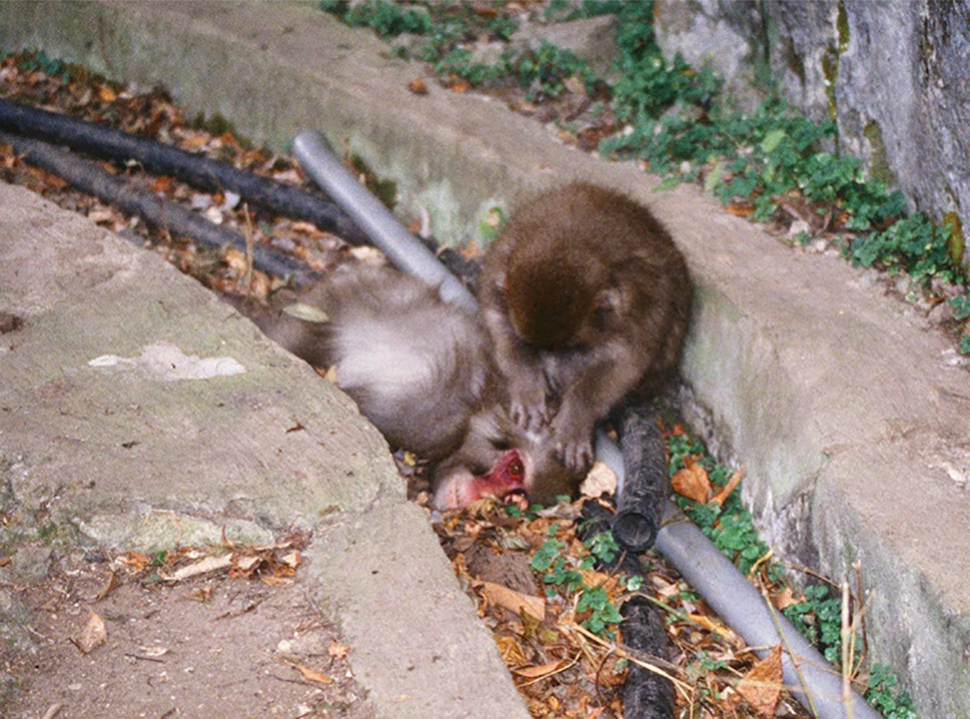
An unrelated 2-year-old female grooming *Pet87*’s corpse (14:17, December 6, 1999). She was a probable grooming partner before *Pet87*’s death, as her mother was one of his confirmed grooming partners

The fourth-ranking male (*Pet87*, formal name *M87 Bera’53′71’*, 12 years old) was found lying dead in a ditch about 2 m above the feeding ground at 14:30 on December 6, 1999 (Fig. 4). M.N. did not notice the corpse earlier despite starting observations at 10:30. *Pet87* was presumed to have died that morning. The day before, he hardly moved while at the feeding ground, and he appeared in poor physical condition (Harada, personal communication). There was no information about how other group members behaved toward him.

For 70 min from finding *Pet87*’s dead body (at 14:30) to the start of provisioning (15:40), at least seven adult females, some immatures and three adult males were observed being within 0.5 m to 5 m from it. One middle-ranking female (*Masia72′78′86*, 13 years old) was the most frequent proximity (within 5 m) partner in the year before his death (December 1998 to November 1999) and third most frequent grooming partner of his nine known adult female partners (Table S3). Most individuals near the corpse sat quietly without showing any overt behavior toward it, but the 2-year-old daughter of *Masia72′78′86* approached and groomed it for 2 min (Fig. 4), then left it calmly. It is unknown whether *Pet87* had an affiliative relationship with this 2-year-old before his death. As Japanese macaque daughters often groom individuals with whom their mothers have grooming interactions (Nakamichi and Yamada 2007), it seems likely that the juvenile female had grooming interactions with *Pet87* before his death.

The lowest ranking (i.e., sixth) central male sat within 2 m of the corpse, the third-ranking male walked past it at a distance of about 1 m, and the alpha male stayed at around 1 m from the corpse for approximately 1 min, foraging in the grass. None of these central males showed any visible response to the corpse, nor were they often seen in proximity to *Pet87* in the year before his death (Table S3). After the group left the feeding site at around 16:20, the corpse was retrieved by park staff. It bore no noticeable wounds except for a 2-cm wound to the scrotum, but it was not festering.

## Discussion

The four cases described here reveal two factors related to who responds to dying and dead conspecifics, and the nature of those responses. First, the external condition of the deceased appears important: no individuals showed avoidance reactions to a corpse showing no obvious external damage, whereas dying and dead individuals that were infested by maggots were clearly avoided. For example, in Case 4 some group members remained near the dead body of an adult male who appeared physically normal, as reported in other provisioned groups of Japanese macaques (Minami and Ishikawa 2023; Mizuhara 1976) and other species where both dying and then dead individuals have been observed (common marmoset, *Callithrix jacchus*, Bezerra et al. 2014; Sichuan snub-nosed monkey, *Rhinopithecus roxellana*, Yang et al. 2016; chimpanzee *Pan troglodytes*, Anderson et al. 2010; van Leeuwen et al. 2016). By contrast, in Cases 1, 2, and 3, when dying individuals were infested by maggots other group members avoided them. In particular, in Case 3, there were two contrasting scenes: upon *Pet79*’s reunion with the group after her temporary disappearance, all of her daughters and her youngest sister groomed her, but about one month later, all avoided her as she was dying and maggot-infested. These three cases suggest a strong aversion to maggots in Japanese macaques, who will not approach even closely affiliated individuals if they show signs of maggot infestation. Watts (2020) reported that only one of 15 chimpanzees who encountered a dead conspecific with swarming flies and maggots touched it, briefly. Avoiding maggot-laden dying or dead conspecifics might decrease the risk of disease and encounters with predators and scavengers, although no studies appear to have been conducted about Japanese macaques’ responses to maggots (for disgust and pathogen avoidance in animals including non-human primates, see Sarabian et al. 2018). In Case 2, any maggots on the *K75*’s body as it lay on the grass were not visible, but no one approached it. This may be because others remembered him with maggots the day before, and his rain-drenched body appearing unusual. In summary, we conclude that Japanese macaques show a clear tendency to avoid dying and dead adult conspecifics that have maggots, whereas fresh corpses showing no visible signs of external damage are not actively avoided; instead, some group members may even behave affiliatively toward such corpses. The literature describing responses in other primate species toward visibly damaged corpses that are maggot-free shows no clear picture (e.g., Barbary macaque *M. sylvanus*, Campbell et al. 2016; chimpanzee, Hosaka et al. 2000). Note that after touching maggots, some monkeys in Cases 1 and 3 sniffed their fingers and then rubbed them on the ground, but it is unclear if their aversion was visually or olfactorily based.

The second factor concerns social affinity with the deceased: individuals who showed positive behaviors to corpses and no aversion were those who had affiliative relationships with the dead individual when alive. In Case 1, only the alpha female groomed around the wound on the back of dying alpha male, and picked up maggots to eat. In Case 2, four adult females crossed the river and remained 10–15 m away from the corpse of the group’s alpha male. One adult female hesitantly approached to within 2–3 m to pick up food, while a 2-year-old female also fed nearby, showing no explicit avoidance. In Case 4, one 2-year-old female groomed the corpse of an adult male. In these examples, all the individuals except for one female in Case 2 had a confirmed or presumed social affinity with the newly deceased individual. These findings are in line with previous studies reporting that social affinity is the primary trigger for positive reactions to dead adult conspecifics (De Marco et al. 2022; Bezerra et al. 2014; Yang et al. 2016; van Leeuwen et al. 2016). However, note that in Case 2 some non-affiliated individuals approached the dead male without showing any sign of aversion, and in Case 3 some individuals with confirmed social affinity with the dying female approached and looked at her but then quickly moved and stayed away. As Minami and Ishikawa (2023) indicated, these findings show that not all affiliated individuals respond positively to the deceased, while some non-affiliated individuals do.

Unlike other individuals who showed aversion toward maggots, alpha female *Pet* picked up maggots and ate them while grooming the dying alpha male, *Rikinio*. Japanese macaques usually eat lice eggs they find while grooming (Tanaka and Takefushi 1993) and grooming-related feeding may be of nutritional benefit (Onishi et al. 2013). Maggots are much larger and thus easier to find and pick up than lice eggs and therefore may have greater nutritional value, but we know of no other reports of Japanese macaques eating maggots. The question remains why *Pet* fed on maggots from *Rikinio*’s back (Case 1) but then 10 years later she did not approach the dying and dead *K75* who had flies with larvae, although she had frequent grooming interactions with him (Case 2).

Some authors suggest that chimpanzees have at least an implicit awareness of death (Anderson et al. 2010; Boesch 2012); however, the picture in monkeys appears less clear. In our Case 4, the lowest ranking central male sat within 2 m of the dead body of the fourth-ranking male, although he usually avoided the latter when he was alive, never previously having been recorded within 2 m of him (see Table S3). Conceivably, the lowest ranking central male considered the completely inert higher-ranking male as no longer needing to be avoided. In Case 2, most group members hesitated to enter the feeding ground where the dead alpha male was lying. Based on these episodes, we cautiously propose that monkeys regard conspecifics lying completely motionless as highly anomalous: what used to be moving (i.e., alive) now no longer moves. This may be a fundamental step in the awareness of death (see Gonçalves and Biro’s (2018) “animacy detection” hypothesis).

To better understand how non-human primates respond behaviorally and emotionally toward dead conspecifics, we need to compile more death-related events from a wider range of species. In particular, quantitative data on social interactions with the deceased before and directly after death are important for answering question about who responds to the death, and how. In addition, quantitative data on individual behaviors such as self-grooming and -scratching, yawning, and activity levels may be useful for evaluating stress following the death of affiliated individuals, particularly in association with physiologic indicators (e.g., hormonal levels) and good qualitative descriptions. The recent demonstration of chimpanzees’ aversion to the odor of putrescine, a major constituent of the “smell of death” (Anderson et al. 2021), indicates the need for further examine of relations between behavioral responses to foul odors from dying and dead individuals, including odor intensity. Such a multi-faceted approach will benefit the continued development of comparative thanatology, the study of how other species perceive and cope with the death of companions and others.

## Supplementary Information

Below is the link to the electronic supplementary material.

Supplementary file1 (PDF 810 KB)

## Data Availability

The data supporting the results of this study are available from the corresponding author upon reasonable request.
