# Supplementary material for: Responses to dying and dead adult companions in a free-ranging, provisioned group of Japanese macaques (Macaca fuscata)
Source: Primates. 2025 Jun 24;66(5):439–48. doi: 10.1007/s10329-025-01196-2 (PMC12391209; doi:10.1007/s10329-025-01196-2)

Supplementary Materials

Nakamichi & Yamada: Responses to dying and dead adult companions in a free-ranging, provisioned group of Japanese macaques (*Macaca fuscata*)

Table S1.

Table S1. Behavior of individuals who were closest to *K75* 's dead body on the morning of August 9, 2003.

Name	Sex	Age	Dominance rank	Kinship with *K75*	Interactions with *K75* for four months befor death		Notes
					Grooming[1)]	Proximity[2)]	
Pet [*, b]	f	32	High	Unrelated	yes[b]	16.4% (8th)	She was around 10 m away from *K75* 's dead body. When soybeans and wheat were scattered near him, she first aproached him, but stopped 2-3 m away, looked at him, then ran away.
Keriia84 [a]	f	19	Middle	Younger sister	no	13.9% (9th)	She was together with her daughter, around 10-15m from the dead body. When soybeans and wheat were scattered near him, she did not approach.
Keriia84'96 [a]	m	7	Middle	Niece	yes	11.9% (10th)	She was together with her mother and 1-year-old daughter, around 10-15 m from *the* dead body.When soybeans and wheat were scattered near him, she did not approach.
Vivia72'83'95 [a]	f	8	Low	Unrelated	no	4.5%	she sat alone and self-groomed around 10-15 m from the dead body. When soybeans and wheat were scattered near him, she did not approach.
Pet88'96 [a]	f	7	Hige	Unrelated	yes	5.0%	When soybeans and wheat were scattered near the dead body, she crossed the river and approached, carrying her baby ventrally. But she came to within around 3 m of the corpse to pick up food, but not closer than that.
Tera68'73'92'01 [b]	f	2	Middle	Unrelated	yes	?	When soybeans and wheat were scattered near the dead body, she crossed the river and approached to within 1 m of it to pick up food. Nine days befor his death (July 31). She was observed to groom and sit in bodily contact with him.
Several (probably 4) juveniles		?	?	?	?	?	When soybeans and wheat were scattered near the dead body, they corssed the river and approached, but never got closer than 5 - 6 m from him.

* *Pet* was the alpha female and *K75* 's most frequent grooming partner during the 4 month period before his death.

[a] These four adult females had already crossed the river and were 10 to 15 m away from *K75* 's dead body in the feeding site, whereas the other group members were still on the other side of the river, 20 m or more away from the dead body.

[1)] Adult individuals who were observed to have grooming interactions with *K75* at least once in 360 20-min grooming sessions conducted during the 4-month-period before his death.

[2)] Adult individuals included in the top 10 animals with the highest frequency of proximity to *K75* during the 4-month-period before his death. Ordinal numbers show the order of the individuals. The number of 20-min proximity sessions conducted during this period was 212. In 201 of these 212 sessions *K75* was observed. For example, *Pet, K75* 's eighth proximity partner, was observed in 33 (16.4%) of the 201.

[b] She had maintained a long-term affiliative relationship with *K75* , who displayed "male care" toward her (holding, carrying, grooming, etc.) for 1.5 years since she was 6 months old. She was observed to groom and sit in bodily contact with him nine days before his death. She was observed tohave grooming interactions or bodily contact with *K75* in 20 (10.0%) of the 201 proximity sessions in which he was observed.

Table S2.

Table S2. The numbers and percentages of grooming interactions between a 27-year-old female, *Pet79* , and adult females adult females during three time phases.

Grooming partners	Sex	Age	Kinship with *Pet79*	During the year preceding her tenporary disappearance (July 2006 to June 2007)[1)]	On the day of her return (July 6, 2007)[2)]	During the 4 weeks following her return (before receiving severe bites) (July 7 to August 2, 2007)[3)]
Pet79'00	f	7	Youngest daughter	131 (56.7%)	Yes	21 (77.8%)
Pet79'98	f	9	Third daughter	49 (21.1%)	Yes	0
Pet79'96	f	11	Second daughter	32 (13.9%)	Yes	1 (3.7%)
Pet79'87	f	20	First daughter	0	Yes	1 (3.7%)
Pet92	f	15	Youngest sister	0	Yes	0
Pet88	f	19	Second youngest daughter	1 (0.4%)	No	0
Other related females*				4 (1.7%)(n=4)	No	0
Unrelated females				14 (8.1%)(n=7)	Yes (n=2)	4 (14.8%)(n=4)
(Total number)				(231)		(27)

1) *Pet79* had grooming interactions with 16 adult females, accounting for 231 (42.5%) of 543 20-min grooming sessions observed during one year before her first temporary disappearance from the group between July 2006 and June 2007.

2) *Pet79* had grooming interactions with 8 adult females for 2 hours of July 6, 2007 directly after her return (*ad libitum* observation).

3) *Pet79* had grooming interactions with 10 adult females, accounting 27 (30.0%) of 90 20-min sessions conducted between July 7 and August 2.

* Females whose blood relatedness to *Pet79* through maternal line was equal to or more than 0.125 were considered as kin, i.e., her daughters, sisters, and nieces.

Table S3.

Table S3. Adult individuals observed to remain or pass within 5 m of *Pet87*'s dead body during a 70-min observation period from author M.N.'s discovery of the body to the start of provisioning in December 6, 1999*

					Behavior and disance to *Pet87*			% of proximity to *Pet87* in the year before his death[1)]		
Adult individuals	Sex	Age	Domi-nance rank	Kinship with *Pet87*	Within 2 m	2-5 m	Behavior when observed	Within 2 m	Within 5 m	Grooming interactions with *Pet87* in the year before his death[2)]
Females										
Beria67'82	f	17	High	Unrelated		○	Sitting without any overt behavior	0	1.6%	No
Beria67'82'91	f	8	High	Unrelated	○		Sitting while auto-grooming	0	1.6%	No
Tera68'73'85	f	14	Middle	Unrelated		○	Sitting without any overt behavior	0.4%	0.8%	No
*Masia72'78'86****	f	13	Middle	Unrelated	○		Sitting without any overt behavior	4.9%	7.8%***	Yes (16.0%)
Masia72'78'91	f	8	Middle	Unrelated	○		Sitting without any overt behavior	0	2.5%	No
Masia72'78'93	f	6	Middle	Unrelated	○		Sitting without any overt behavior	0	0.0%	No
Rolina81	f	18	Low	Unrelated	○		Sitting without any overt behavior	3.3%	3.3%	Yes (4.0%)
Males										
Keriia75	m	24	First**	Unrelated	○		Foraging in the grass for 1 min	0	1.2%	No
Pet90	m	9	Third**	Unrelated	○		Passing the corpse next to 1 m	0.8%	0.8%	No
Rolia68'77	m	22	Sixth**	Unrelated	○		Sitting without any overt behavior	0	0.4%	No

* Observations were made for 70 min from finding *Pet87*'s dead body (14:30) to the start of provisioning (15:40).

** Dominance rank among six central males.

*** *Masia72'78'86* was the most frequent proximity and the third most frequent grooming partner of *Pet87* in the year before his death.

1) Proximity (within 2 m and 5 m) of each individual was calculated as percentage of 243 20-min proximity sessions in which *Pet87* was observed in the year before his death from December 1998 to November 1999.

2) *Pet87* was observed to have grooming interactions with adult individuals in 25 of 288 20-min grooming sessions conducted in the year before his death. Values in parentheses show percentage of his 25 grooming interactions.

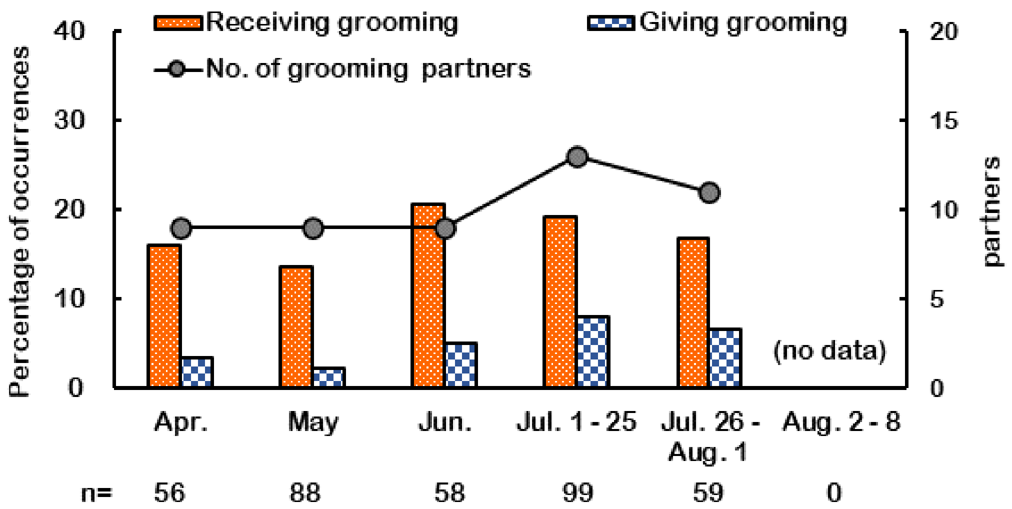

Fig. S1. Percentage of 20-min sessions in which *K75* groomed or received grooming from adults, and number of adult grooming partners in the 4 months before his death (August 9, 2003). Observations were made by author M.N. at the feeding site during 56, 88, 58, 99, and 59 sessions respectively, from left. See Nakamichi and Shizawa (2003) for further details on observational methods.

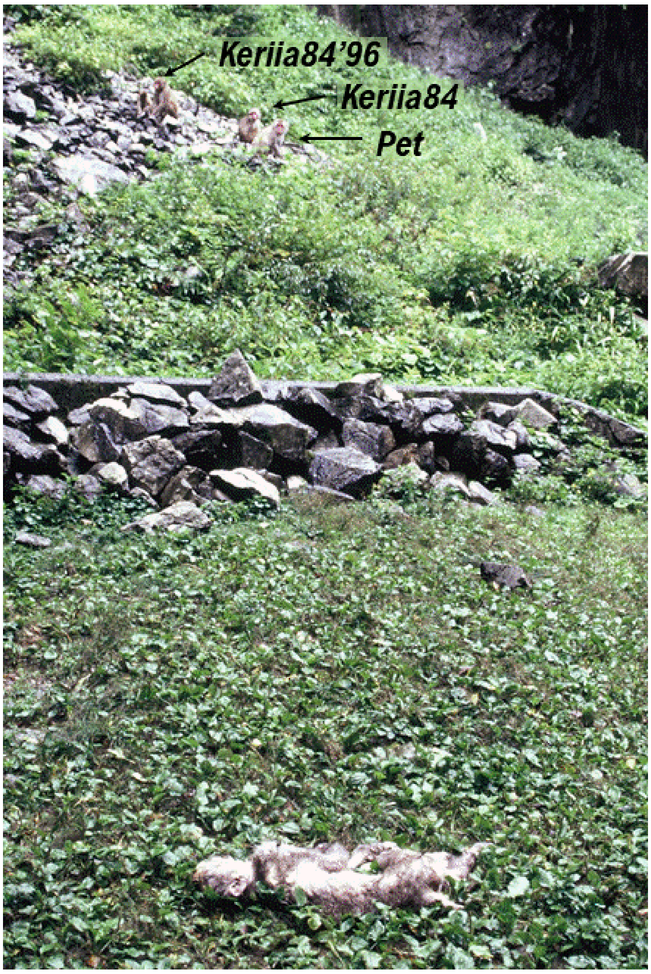

Fig. S2. *K75*'s dead body lying on the feeding ground. No monkeys were immediately nearby when it was found, although the group had already visited the feeding site. Three adult females, the 32-year-old alpha female *Pet* (top right), *K75*'s youngest sister (*Keriia84*, 19 years old, top middle) and her adult daughter (*K75*'s niece, *Keriia84'96*, 7 years old) with her 1-year-old juvenile female (*K75*'s grandniece) were the nearest, around 15 m away. *Pet* was his most frequent grooming partner and eighth most frequent proximity partner (within 5 m) in the 4 months prior to his death. *Keriia84* was not a grooming partner but was his ninth proximity partner, while *Keriia84'96* was one of his 24 adult grooming partners, and tenth most frequent proximity partner. (Photo originally appeared in Nakamichi, 2019)

Fig. S3. A 2-year-old unrelated female (*Tera68'73'92'01*) picks up wheat within 1 m of *K75*'s corpse, showing no signs of aversion. She had a long-term affiliative relationship with *K75* which included being held and carried, grooming, and physical contact ("male care") for 1.5 years until his death (08:08, August 9, 2003).
(Photo originally appeared in Nakamichi, 2019).

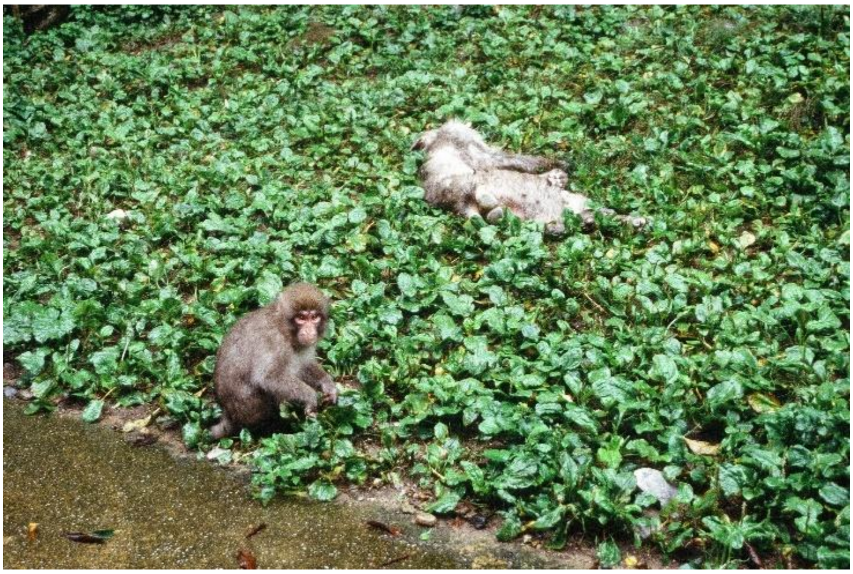

Fig. S4. After *K75*'s corpse was removed by park staff at around 08:40, feeding began and some monkeys picked up wheat about 1 to 2 m away from where the corpse had lain, but not in the specific place where it had been (indicated by a light brown circle).

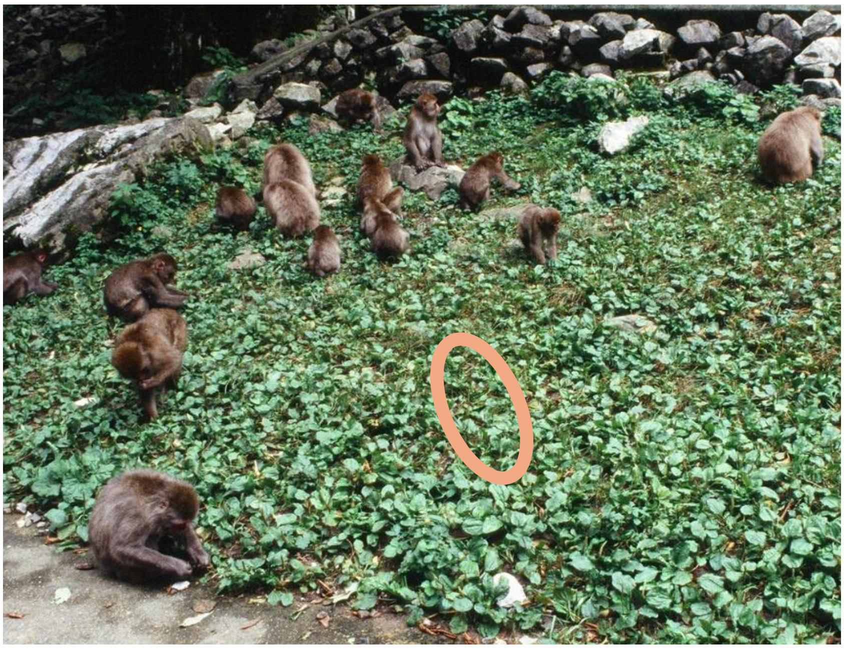

Supplement: Supplementary file 1 — Supplementary file1 (PDF 810 KB) [file 10329_2025_1196_MOESM1_ESM.pdf]
